# Application of Grey Relational Analysis to Predict Dementia Tendency by Cognitive Function, Sleep Disturbances, and Health Conditions of Diabetic Patients

**DOI:** 10.3390/brainsci12121642

**Published:** 2022-11-30

**Authors:** Chiung-Yu Huang, Yu-Ching Lin, Yung-Chuan Lu, Chun-I Chen

**Affiliations:** 1 Nursing Department, I-Shou University, Kaohsiung 82445, Taiwan; 2 Department of Family Medicine and Physical Examination, E-Da Hospital, Kaohsiung 82445, Taiwan; 3 College of Medicine, School of Medicine for International Students, I-Shou University, Kaohsiung 82445, Taiwan; 4 Division of Endocrinology and Metabolism, Department of Internal Medicine, E-Da Hospital, Kaohsiung 82445, Taiwan; 5 Management College, I-Shou University, Kaohsiung 82445, Taiwan

**Keywords:** dementia, sleep quality, depressive symptoms, cognitive function, quality of life, grey relational analysis

## Abstract

***Background***: The number of elderly diabetic patients has been increasing recently, and these patients have a higher morbidity of dementia than those without diabetes. Diabetes is associated with an increased risk for the development of dementia in elderly individuals, which is a serious health problem. ***Objectives***: The primary aim was to examine whether diabetes is a risk factor for dementia among elderly individuals. The secondary aim was to apply grey theory to integrate the results and how they relate to cognitive impairments in elderly diabetic patients and to predict which participants are at high risk of developing dementia. ***Methods***: Two hundred and twenty patients aged 50 years or older who were diagnosed with diabetes mellitus were recruited. Information on demographics, disease characteristics, activities of daily living, Mini Mental State Examination, sleep quality, depressive symptoms, and health-related quality of life was collected via questionnaires. The grey relational analysis approach was applied to evaluate the relationship between the results and health outcomes. ***Results***: A total of 13.6% of participants had cognitive disturbances, of whom 1.4% had severe cognitive dysfunction. However, with regard to sleep disorders, 56.4% had sleep disturbances of varying degrees from light to severe. Further investigation is needed to address this problem. A higher prevalence of sleep disturbances among diabetic patients translates to a higher degree of depressive symptoms and a worse physical and mental health-related quality of life. Furthermore, based on the grey relational analysis, the grey relation coefficient varies from 0.6217~0.7540. Among the subjects, Participant 101 had the highest value, suggesting a need for immediate medical care. In this study, we observed that 20% of the total participants, for whom the grey relation coefficient was 0.6730, needed further and immediate medical care.

## 1. Introduction

Diabetes, the fifth highest cause of death reported in 2018, is highly prevalent, with an estimated 642 million people predicted to have diabetes by 2040 [1]. The incidence of diabetes has also been growing rapidly in elderly populations, in which diabetes is strongly associated with dementia. Diabetes is associated with an increased risk for the development of both vascular dementia and nonvascular dementia in elderly individuals [2,3]. In general, patients with diabetes have a 1.6 to 2 times higher rate of dementia than the average person [4]. Type 2 diabetes is a highly prevalent chronic disease among elderly individuals and is also a risk factor for dementia. Additionally, diabetes among elderly patients has become a serious problem and can be a burden for patients, families, and society. Therefore, the prevention of diabetes comorbidity is becoming more important.

There is a relationship between diabetes and cognitive dysfunction, and factors such as abnormal glucose metabolism, insulin deficiency, insulin antagonists, or abnormal protein build-up in the brain [5] may be the cause of this cognitive dysfunction [6]. Quite a few studies have mentioned the strong relationships between diabetes and Alzheimer’s disease (AD) [7,8,9], but some results are inconsistent [10,11,12]. Ott et al. [7] found that diabetes mellitus almost doubled the risk of dementia. Schrijvers et al. [8] proposed that levels of insulin and insulin resistance were associated with a higher risk of AD within 3 years of baseline. Xu et al. [9] concluded that diabetes mellitus increases the risk of dementia, specifically vascular dementia, in very old people. Akomolafe et al. [10] reported that diabetes mellitus did not increase the risk of incident AD in the Framingham cohort overall; however, DM may be a risk factor for AD in the absence of other known major AD risk factors. Euser et al. [11] suggested that elevated fasting glucose levels and insulin resistance are not associated with worse cognitive function in older people without a history of diabetes. MacKnight, Rockwood, and McDowell [12] could not find substantial evidence to prove an association between diabetes and incident Alzheimer’s disease, even though diabetes was associated with incident vascular cognitive impairment. Hyperglycemia induced by diabetes may cause cognitive decline, although the mechanism has not been proven. As good blood sugar control reduces complications and decreases cognitive impairments in diabetic patients, well-controlled glycemia is important for preventing dementia [13,14]. The detection of moderate cognitive dysfunction and administration of timely preventive measures and treatments in the early stages of dementia can reduce the risk factors for dementia caused by the blockage of blood vessels, which will greatly decrease the incidence of dementia and thus reduce the medical and economic burden on families.

In Taiwan, the prevalence of diabetes among individuals aged 18 and over is 11.8% (13.1% for men and 10.5% for women), and there are an estimated 2.28 million diabetic patients and 25,000 new cases per year. The prevalence rates of diabetes among elderly men and women are 27.7% and 24%, respectively, showing that the incidence tends to increase with age in both sexes [1]. Complications of diabetes, including cerebrovascular disease, cardiovascular disease, kidney disease, the terminal stage of neuropathy, and retinopathy [15], not only impact the quality of life of diabetic patients but also consequently increase medical costs, accounting for more than one-eighth of the total cost of health care [16].

The results of the Ministry of Health and Welfare’s epidemiological survey of dementia in 2011 and demographic information from 2017 estimated that there were 3,192,477 people aged 65 years and older in Taiwan, of whom 586,068 (18.36%) had mild cognitive impairments. The number of people with dementia was 253,511, or 7.94%; this included 102,926 (3.22%) people with mild or worse dementia and 150,585 (4.72%) people with mild or worse dementia, showing that 1 in 13 people over 65 years of age had dementia and that 1 in 5 people over 80 years of age was mentally impaired. In addition, poor glucose control for diabetic patients may consequently impact complications and thus increase the risk of dementia. Although the Ministry of Health and the medical profession are beginning to pay attention to this problem, we should be more active in preventing this serious health condition and developing more effective preventive strategies for dementia risk factors.

Since dementia causes a tremendous social burden, the prediction of dementia in individuals with diabetes is important to prevent or treat early-stage dementia. In recent years, many studies have been devoted to exploring prediction models. Lieza et al. [17] adopted two longitudinal cohorts of T2DM patients aged 60+ with ten years of follow-up to create and validate the risk score. This is the first risk score capable of predicting a 10-year individualized dementia risk in patients with T2DM. Chloe et al. [18] investigated the relationship between the diabetes-specific dementia risk score (DSDRS) and concurrent and future cognitive impairment (CI) in type 2 diabetes (T2D). They showed that higher DSDRS was associated with a higher probability of CI at baseline and follow-up. Omar et al. [19] also adopted the DSDRS to predict cognitive performance risk. These findings support that T2D-related factors have a significant burden on functional status, quality of life, disability, and dementia risk. Many studies [20,21,22] have used artificial intelligence algorithms to predict the onset of dementia. In this study, we tried to propose a novel prediction method, the grey model, to identify the possibility of dementia in diabetic patients. The merit of the grey model is to analyze uncertain and incomplete information. This pioneering research hopes to introduce a novel methodology for doctors or healthcare professionals to predict the possibility of dementia from the biomarkers of DM patients.

The relationship between cognition and the duration of diabetes among elderly individuals is unclear. The primary aim of this study was to examine whether incident diabetes is a risk factor for dementia in elderly individuals and to clarify the relationships among diabetes characteristics, activities of daily living, cognitive function, sleep quality, depressive symptoms, and health-related quality of life. The secondary aim was to use grey theory to integrate the results and how they relate to cognitive impairments in elderly diabetic patients and to predict which participants are at high risk of developing dementia.

The grey relational theory was proposed by Deng [23] and designed to analyze uncertain and incomplete information systems under the condition of few collected data. Moreover, grey relational theory can be used to identify the relationship among mutually influencing factors. To date, it has been widely applied in many fields. Canbolat et al. [24] studied the performance optimization of absorption refrigeration systems using Taguchi, ANOVA, and grey relational analysis methods. Yazdani et al. [25] applied grey relational analysis to the fuzzy multi-attribute decision framework with the integration of quality function deployment (QFD). Liu et al. [26] combined grey relational analysis and failure mode and effect analysis in risk management. However, the application of grey relational theory is seldom applied in the field of medical diagnosis. In this study, the research team adopted grey relational analysis to integrate the questionnaire results to fulfill the idea of comprehensive and intelligent diagnosis.

The relationship between diabetes duration and cognitive impairments in elderly patients is unclear. Our aims are as follows: (1) The primary aim was to examine whether diabetes duration is a risk factor for dementia in elderly diabetic individuals and clarify the relationships among diabetes characteristics, activities of daily living, cognitive function, sleep quality, depressive symptoms, and health-related quality of life. (2) The secondary aim was to use grey theory to integrate the investigation results and study how they relate to cognitive impairments in elderly diabetic patients.

## 2. Methods

### 2.1. Study Design

A predictive study design was used to conduct a cross-sectional survey between September 2020 and March 2021. A questionnaire developed from previous studies and validated by expert review was used to investigate the relationships among demographic factors, disease characteristics, depressive symptoms, and health-related quality of life. The attrition rate was 96.8%.

### 2.2. Participants

A convenience sample of outpatients was recruited from two metabolic departments at two general hospitals in the southern region of Taiwan. The eligibility criteria were as follows: (1) patients diagnosed with type 2 diabetes by a physician; (2) adults over 50 years of age; and (3) patients with no mental disorders. The exclusion criteria included a history of depression or psychosis and treatment with psychiatric medication.

### 2.3. Setting and Sampling

Permission was obtained from the Institutional Review Boards of the participating hospitals. The study design was used to improve generalizability by selecting eligible subjects from the diabetic population. To determine an adequate sample size, the significance level of α, effect size, and power were estimated. Cohen [27] suggested that a medium effect size was conservative without being excessively stringent. A conservative effect size of 0.15, as suggested by Cohen [27], was used for this study. Accordingly, a sufficient sample size was calculated as 179 for this proposed study. The sample size was calculated as follows: N = [L/γ] + k + 1 [27]. For α = 0.05 and a power of 0.80 with 20 predictors for testing, the value of Lambda (L) was determined to be 23.7 (L = table value for a specified α and power) according to the power table provided by Cohen [27]. In addition, this study used correlation and simple and multiple regression. The calculation was as follows: N = [23.7/0.15] + 20 + 1 = 179. Considering that missing data might occur during data collection, the investigator increased the number to 220 for recruitment.

### 2.4. Variables and Research Instruments

The measures consisted of demographic information, disease characteristics (including physiological indicators), sleep quality, activity of daily living, Mini-Mental State Examination, Geriatric Depression Scale, and a health-related quality of life scale as follows:

Disease characteristics included duration of diabetes, numbers of complications of diabetes, fasting glucose, and glycated hemoglobin (HbA_1_C); demographic information included age, sex, household monthly income reported in New Taiwan currency (NT dollars: <25,000, 25,001–50,000, 50,001–75,000, >75,000), marital status (single, divorced or separated, married or cohabiting, widowed), and education (illiteracy, elementary, junior high school, senior high school, and college or above). Cholesterol, high-density lipoprotein cholesterol (HDL-C), low-density lipoprotein cholesterol (LDL-C), triglycerides (TGs), types of chronic diseases (hypertension, heart disease, hyperlipidemia, atherosclerosis, atrial fibrillation, etc.), weight, height, abdominal circumference, amount of exercise per day, exercise frequency, smoking habit, alcohol consumption, complications, and diabetes treatment (oral or insulin injection) were all collected.

### 2.5. Pittsburgh Sleep Quality Index (PSQI)

The PSQI was implemented to evaluate sleep quality for diabetic patients and was developed by Buysse, Reynolds, Monk, Berman, and Kupfer [28]. The PSQI, which includes 19 items, is divided into seven sections to measure participants’ viewpoints of sleep quality. All sections are subjective: sleep quality, latency, duration, sleep efficiency, sleep disturbances, the use of sleep aids (pills), and daytime dysfunction over the last month. The global PSQI score of sleep quality ranges from 0 to 21, with higher scores indicating worse sleep quality. A PSQI score greater than 5 was defined as sleep disturbance (SD) [28]. Cronbach’s α was 0.82 in the study by Kao et al. [29] and 0.81 in this study.

### 2.6. Activity of Daily Living

The Barthel Index is a daily function assessment scale used to evaluate individuals’ physical function [30]. This index has been widely used in the field of rehabilitation and with elderly patients, mainly to measure the effectiveness of treatment and degeneration of patients. This is a 10-item scale with a total score between 0 and 100 that is used to measure activities of daily living, with higher scores indicating greater independence. In this study, the Cronbach’s alpha for the Barthel scale was computed to be 0.89 [31].

### 2.7. Mini Mental State Examination (MMSE)

The Mini-Mental State Examination (MMSE), developed by Folstein and Mc Huge [32], includes sense of orientation, attention, memory, language, oral understanding and behavior, construction, and other items. The evaluation process has no time limit. A total score of 30 points is possible, and a higher score indicates better cognitive function. A total score of <24 points indicates that the individual has mild cognitive impairment, and <16 points indicates severe cognitive impairment. Scores in the range of 24–30 are classified as cognitive function completeness; those in the range of 18–23, mild cognitive impairment; and those in the range of 0–17, severe cognitive dysfunction. The MMSE is widely used in medical community mental assessments.

### 2.8. Geriatric Depression Scale

Sheikh and Yesavage simplified the Geriatric Depression Scale to the GDS-SF15 based on the original Geriatric Depression Scale developed in 1982 [33], a 15-question self-completed assessment scale for screening depression tendencies in elderly people. This questionnaire mainly focuses on items related to mental symptoms, such as sadness, apathy, and boredom, to reduce the fatigue of elderly individuals when filling out the questionnaire. The items in this questionnaire require “yes/no” answers. Of a total score of 15 points, <4 means no depressive tendency, 5–10 shows a slight depressive tendency, and 10 to 15 points indicates a more severe depressive tendency. A higher score indicates a higher tendency for depression, and Huang, Hsieh, and Lai’s [34] Chinese version of the scale was used to test the effectiveness of music and relaxation intervention in the elderly population.

### 2.9. Health-Related QOL

We applied the 36-item Short-Form Health Survey as a generic indicator of health. This survey measures HRQOL and includes eight subscales that are applicable to the general health of individuals, comprising physical function, role limitations, bodily pain, social function, general mental health, vitality, energy or fatigue, and general health perceptions. HRQOL is typically divided into a physical QOL (PQOL) component and mental QOL (MQOL) component, which were used in this study. The Cronbach’s alpha values in our study ranged from 0.76 to 0.84 among these subscales.

### 2.10. Ethical Considerations

We received approval from the Institutional Review Board of the general hospital. Endocrinologists referred eligible participants to the investigator. Participants were provided information about the purpose, benefits, risks, and data collection procedures of the current study. All information was provided in both verbal and written form, and participants could drop out of the study at any time. After receiving written informed consent from the participants, the investigator performed a face-to-face interview. Studies were conducted between September 2020 and March 2021. We maintained privacy and confidentiality for each participant and assigned each participant a code number; only the researchers had access to the data.

### 2.11. Data Analysis

After coding the valid questionnaires in this study, the data were entered into the computer in the Statistics Package for Social Science for Windows 20.0 software package. The statistical analysis methods applied were descriptive statistics and Pearson’s correlation statistical methods. Preliminary data analysis included a description of the sample using frequency distributions for categorical variables and descriptive statistics. Pearson’s correlation was used to evaluate the degrees and strengths of the relationships among the study variables. A *p* value of 0.05 indicated statistical significance.

### 2.12. Grey Relational Analysis (GRA)

In this study, grey relational analysis was adopted to provide physicians with an integrated diagnosis of biological indexes and measured instrument results. Each measurement tool of the physiological questionnaire provides physicians only with individual judgments of various corresponding symptoms; for example, the PSQI is for sleep quality, and the MMSE is for cognitive function. However, there is no comprehensive evaluation method for considering all measurement results. In this study, grey relational analysis was introduced to consider all measurement tools and provide a comprehensive diagnostic basis. The reference series *x*_0_, which is composed of the measured results of all instrument tools, was calculated by grey relational analysis, and the comparison series *x_i_*s was collected from each participant. The grey relation coefficient, γ, between the reference series and comparison series was calculated by grey relational analysis. If γ(*x*_0_,*x_i_*) ≧ γ(*x*_0_,*x_j_*), *x_i_* has a greater relationship with *x*_0_ than *x_j_*, that is, *x_i_* > *x_j_* [35].

A local GRA was adopted in this study, and the derivation procedures are as follows:

Step 1: A reference sequence, *x*_0_, which was extracted from the collected biological data and measured questionnaires of each participant, and a comparative sequence, *x_i_*, were established:

(1) $$x_{0}(k)=x_{0}(1),x_{0}(2),\ldots x_{0}(n)$$

(2) $$x_{i}(k)=x_{i}(1),x_{i}(2),\ldots x_{i}(n)$$

where  k=1, 2, 3,⋯, n, i=1, 2, 3,⋯, m. *n* is the number of biological indexes and measured questionnaires. m is the number of participants. $x_{0}(k)$ is a reference sequence, and $x_{i}(k)$ is a comparative sequence.

Step 2: The original data were standardized by the initial value technique:

(3) $$\mathrm{Initial}\;\mathrm{value}\text{:}\;x_{i}(k)=\frac{x_{i}(k)}{x_{i}(1)}$$

Step 3: The grey relational coefficient was obtained used the following equation:

(4) $$\gamma (x_{0}(k)\;,\;x_{i}(k))=\frac{\Delta \min+\zeta \Delta \max}{\Delta_{oi}(k)+\zeta \Delta \max}$$

where  k=1, 2, 3,⋯, n, i=1, 2, 3,⋯, m, and γ is the grey relational coefficient. $\Delta_{oi}=||x_{0}(k)-x_{i}(k)||\;$ is the absolute value of the kth difference between $x_{0}$ and $x_{i}$, $\Delta_{\min.}=\underset{i}{\min}\;\underset{k}{\min}\Delta_{oi}$, $\Delta_{\max.}=\underset{i}{\max}\;\underset{k}{\max}\Delta_{oi}$.

ζ: distinguishing coefficient; ζ∈ [0,1]; in general, the distinguishing coefficient is 0.5 and can be adjusted as needed. The adjusted value will change only the relative value and will not affect the order of the grey relational grade [36].

Step 4: The grey relational grade was obtained using the mean value of the grey relational coefficient:

(5) $$\gamma (x_{0}\;,\;\;x_{i})=\frac{\;1\;}{n}\sum_{k=1}^{n}\gamma (x_{0}(k)\;,\;\;x_{i}(k))$$

The closer the value of the grey relational grade is to unity, the higher the relational grade of the reference sequence; otherwise, it is lower.

Step 5: The grey relational order was obtained.

The grey relational grade indicates the relational grade between each sequence and the reference sequence. The order is determined by ranking the relational grades from the highest value to the lowest, which is called the grey relational order. It means that the relational grade between *x_i_* and *x*_0_ is greater than that between *x_j_* and *x*_0_, that is, *x_i_* is more similar to *x*_0_.

## 3. Results

### 3.1. Participant Characteristics

We recruited 220 patients with diabetes mellitus between the ages of 51 and 89 (M = 66.6, SD = 7.6) from outpatient departments in two urban hospitals in southern Taiwan. The majority of participants were male (52.7%), approximately 61% of the participants (or less) had a high school diploma, and the majority of patients (47.3%) had a household income less than TWD 25,000 per month. The duration of diabetes varied from 4 months to 420 months (M = 136.3); two patients had a duration of 4 months and one patient had a duration of 420 months, which could be treated as outliers. Most patients had a uniform duration of the disease. Approximately 89.5% of the participants had at least one complication of diabetes. Participants’ fasting glucose levels ranged from 58 to 557 mg/L (M = 140.7), glycated hemoglobin (HbA_1_C) ranged from 5.4% to 12.7% (M = 7.4%, SD = 1.2%) (Table 1), mean body mass index (BMI) was 25.9, triglycerides (TGs) ranged from 39~991 (M = 157.88, SD = 123.04), mean high-density lipoprotein cholesterol level was 49.66, and mean low-density lipoprotein cholesterol level was 84.01.

Geriatric depression symptom (GDS) scores of 0 to 4 indicate no symptoms of depression, scores of 5 to 10 indicate mild depressive symptoms, and scores of 10 to 15 indicate severe depressive symptoms. Only 13.6% of the participants had depression: 12.2% had mild depression, and 1.4% had moderate to severe depression. Depression status scores ranged from 0 to 13 (M = 2.6, SD = 2.4). On the MMSE, 13.6% of patients with diabetes had mild and moderate cognitive impairments, and most (86.4%) participants had normal cognitive function (Table 1).

### 3.2. Relationships between Variables

In this research, the variables collected were tested and complied with normality. Table 2 shows that age was negatively correlated with education (*r* = −0.42, *p* < 0.01), income (*r* = −0.17, *p* < 0.05), and MMSE score (*r* = −0.33, *p* < 0.01). In addition, age was negatively correlated with fasting glucose (r = −0.15, *p* < 0.05) and positively correlated with diabetes duration (*r* = 0.17, *p* < 0.05). There was a significant positive correlation between education and income (*r* = 0.28, *p* < 0.01), MMSE score (*r* = 0.45, *p* < 0.01), and MQOL and PQOL (*r* = 0.15, *p* < 0.05; *r* = 0.20, *p* < 0.01). There was a significant positive correlation between the duration of diabetes and HbA_1_C (*r* = 0.26, *p* < 0.01). Household monthly income was significantly positively correlated with MMSE score (*r* = 0.27, *p* < 0.01). Glycemic control (fasting glucose and HbA_1_C) was not significantly related to DSs, SQ (sleep quality), MQOL, or PQOL; however, glycemic control was significantly positively correlated with triglycerides (TGs). Total cholesterol was positively correlated with LDL-C and TG; HDL-C was negatively correlated with HbA_1_C and fasting sugar. TG was significantly positively related to GDS, and GDS had a strong negative relationship with MQOL and PQOL and a significant positive relationship with SQ (*r* = −0.68, *p* < 0.01; *r* = −0.49, *p* < 0.01; *r* = 0.40, *p* < 0.01). In addition, SQ had a significantly negative correlation with PQOL and MQOL (*r* = −0.33, *p* < 0.01; *r* = −0.38, *p* < 0.01).

### 3.3. Grey Relational Analysis

In this study, data on BMI, HbA_1_C, fasting sugar, total cholesterol, HDL-C, LDL-C, TG, SQ, MMSE, GDSs, PQOL, and MQOL were collected for further grey relational analysis. The results of the grey relational analysis are shown in Table 3 (complete grey relational analysis information is provided in the Supplemental data Table S1). The results could be used as an artificial intelligence basis of diagnosis. The grey relational coefficients of all participants ranged from 0.6217 to 0.7540. Participant 101 had the highest probability score, which meant that he or she was the most likely to have Alzheimer’s disease among all the participants. Our research team recommended immediate medical care before further deterioration. Medical care could be administered as medication or cognitive behavioral treatment. In this study, based on the Pareto theorem, which is a theory maintaining that 80 percent of the output from a given situation or system is determined by 20 percent of the input, we suggested that 20% of all participants needed further medical care. The corresponding grey coefficient was 0.6730.

## 4. Discussion

### 4.1. Study Variables

There was no direct association between sleep quality or sleep segments and the lipid profile status of the patients, including triglyceride level, total cholesterol, low-density lipoprotein cholesterol (LDL-C), and high-density lipoprotein cholesterol (HDL-C) in this study. However, there were significant relationships between SQ and mental and physical QOL; SQ was significantly related to mental and physical QOL in diabetes patients. We consider that there may be some connections between variables that need further study.

### 4.2. Glycemic Control and Dementia

According to β-amyloid peptide accumulations, which have been considered to be the fundamental cause of AD (Alzheimer’s disease), the occurrence of diabetes is related to insulin resistance and hyperinsulinemia and might interfere with β-amyloid peptide metabolism. Additionally, insulin can cross the blood–brain barrier, and insulin levels in the brain are initially higher and then downregulated in diabetic patients. In our study, 86% of the participants had normal MMSE scores, and 86% had normal GDS scores. It might be that most participants’ blood sugar was well controlled. Only 15% of DM patients had mild to severe MMSE scores, but we found that depressive symptoms were significantly associated with MMSE scores and TGs, which might be related to dementia after a longer diabetes duration in the future. Because a previous study mentioned that diabetes has been reported to be associated with AD [5,6], healthcare professionals can use therapeutic strategies for DM as an important measure for preventing AD development.

The relationship between DM and AD has been noted in recent years. In our research, 15% of DM patients were found to have abnormal MMSE scores, which require more attention about the risk of dementia. Compared to a previous study, DM almost doubled the risk of dementia in elderly subjects after an average 2-year follow-up [3]. Since DM at baseline was associated with incident vascular cognitive impairment but not AD in many studies, the focus on the chronological evolution to AD is an important issue for healthcare professionals. In modern medicine, there is no solid evidence to prove the relationship between DM and AD. In this study, the grey relational analysis is proposed to be the pioneer study for predicting AD.

### 4.3. Sleep Quality and Glycemic Control

According to Pearson’s correlation, there were no direct associations between sleep quality and glycemic control. However, fasting sugar and HbA_1_C were both significantly positively correlated with TG.

The duration of diabetes may play an important role in AD pathogenesis and lower MMSE scores; however, there was no significant association between MMSE scores and the duration of diabetes mellitus in our study, which might be because the duration of diabetes was not long enough to reflect the progression of AD. However, this risk factor should be studied further.

### 4.4. Sleep Quality, GDS, and QOL

Consistent with a previous study [29,37], we found that the poorer the sleep quality was, the poorer the HRQOL of the participants. Additionally, the poorer the sleep quality of diabetic patients was, the more depressive symptoms they had [38]. Sleep disturbances are usually comorbid with health problems of individuals of various ages or with chronic diseases; thus, healthcare professionals should pay more attention to enhancing sleep quality. However, a previous study showed that poor sleep quality may result in unsatisfactory glycemic control and consequently induce health problems; nevertheless, inconsistent with our findings, we might need a longer period of observation.

## 5. Limitations

There were a few limitations of the study. First, the participants were all aged 50 years or older, which may impact the results. Huang et al. [3] mentioned one study that recruited DM patients 55 years or older as subjects for DM related to AD. These recruitment criteria for age are close to those of the present study. The other important reason for recruiting younger patients is that the evolution from normal to AD takes time. To fulfill one of the study purposes, to examine whether incident diabetes is a risk factor for AD, the definition of recruitment criteria is crucial for this study. Second, for future studies, the design should be longitudinal to observe and compare the progression of AD in diabetic patients over a longer period of time. Third, a longer follow-up may yield significant findings for the HRQOL of patients with diabetes-related AD. Fourth, the MMSE is adopted as a tool to judge the cognitive function of DM patients by its score and is not used to confirm or exclude dementia.

## 6. Conclusions

This is the first study to use grey theory to predict cognitive function, depressive symptoms, and health-related quality of life by applying fasting glucose, HbA_1_C, BMI, and biological markers in patients with diabetes in Taiwan. More than half of the participants had sleep disturbances of varying degrees from light to severe. Further tracking is needed to solve this problem and prevent complications for diabetic patients. The more sleep disturbances there are in diabetic patients, the higher the degree of depressive symptoms and the worse their physical and mental health-related quality of life. Furthermore, based on grey relational analysis, the grey relation coefficient varied from 0.6217~0.7540. In this study, we proposed that 20% of the total participants, of whom the grey relation coefficient was 0.6730, needed further and immediate medical care.

## Figures and Tables

**Table 1:** Sample characteristics (*n* = 220).

Variables	Mean	SD	Range
Age	66.6	7.6	51–89
Duration of diabetes (months)	136.3	99.1	4–420
Glycated hemoglobin (HbA1C, %)	7.4	1.2	5.4–12.7
Fasting sugar (mg/dL)	140.7	58.8	58–557
Body mass index (BMI, kg/m^2^)	25.9	3.8	18.4–40.0
Triglyceride (TG, mg/dL)	157.9	123.0	39–991
Total cholesterol (mg/dL)	162.3	46.7	34–480
High density lipoprotein-cholesterol (HDL-C, mg/dL)	49.7	12.9	27–108
Low density lipoprotein-cholesterol (LDL-C, mg/dL)	84.0	26.1	11–180
Mini-Mental State Examination (MMSE)	27.2	3.3	12–30
Activity of daily living (ADL)	99.6	2.7	70–100
Geriatric depressive symptoms (GDS)	2.6	2.4	0–13
Physical quality of life (PQOL)	74.5	18.8	13–99
Mental quality of life (MQOL)	79.5	12.4	28–95
Sleep quality (SQ)	6.7	3.3	1–17
	*n*	%	
Gender			
Male	116	52.7	
Female	104	47.3	
Marital status			
Single	9	4.1	
Separated/divorced	16	7.3	
Widowed	40	18.2	
Married	155	70.5	
Education (school years)			
0	10	4.5	
≦6	83	37.7	
≦6–9	42	19.1	
≦9–12	76	34.5	
>12	9	4.2	
Household income (TWD)			
<25,000	104	47.3	
25,001–50,000	72	32.7	
50,001–75,000	24	10.9	
75,001–100,000	9	4.1	
>100,000	11	5.0	
Complications			
0	23	10.5	
≧1	197	89.5	
MMSE			
Severe	4	1.8	
Mild	26	11.8	
Normal	190	86.4	
GDS			
None	190	86.4	
Mild	27	12.2	
Severe	3	1.4	

**Table 2:** Correlations among independent variables, depressive symptoms, and quality of life.

Variables	1	2	3	4	5	6	7	8	9	10	11	12	13	14	15	16	17	18
1. Age	1																	
2. Education	−0.42 **	1																
3. Duration	0.17 *	−0.05	1															
4. Income	−0.17 *	0.28 **	0.01	1														
5. BMI	−0.1	0.00	−0.09	0.00	1													
6. Fasting sugar	−0.15 *	−0.05	−0.01	0.03	0.06	1												
7. HbA_1_C	−0.12	−0.09	0.26 **	0.02	0.13	0.57 **	1											
8. Complications	0.05	−0.13	0.06	−0.08	0.14 *	0.08	0.02	1										
9. TG	−0.10	0.07	−0.02	−0.01	0.01	0.26 **	0.17 *	0.01	1									
10. TChol	−0.01	0.03	0.01	0.02	0.01	0.07	0.06	0.11	0.49 **	1								
11. HDL-C	−0.03	0.07	0.00	0.16 *	−0.10	−0.18 **	−0.14 *	−0.11	−0.33 **	0.12	1							
12. LDL-C	0.10	0.00	−0.06	0.04	−0.03	−0.05	−0.11	−0.09	0.10	0.50 **	0.12	1						
13. MMSE	−0.33 **	0.45 **	0.04	0.27 **	0.14 *	0.16 *	0.09	0.02	0.02	0.08	0.05	0.07	1					
14. ADL	−0.12	0.03	−0.10	−0.08	0.06	0.06	0.09	−0.05	−0.04	−0.01	0.13	−0.10	0.07	1				
15. GDSs	0.07	−0.09	0.06	−0.06	−0.04	0.04	−0.04	−0.00	0.19 **	0.10	−0.01	0.02	−0.14 *	−0.08	1			
16. SQ	−0.00	−0.06	0.03	−0.00	0.01	0.02	−0.02	0.03	−0.00	−0.06	0.07	−0.06	0.07	0.02	0.40 **	1		
17. MQOL	−0.05	0.15 *	−0.04	−0.08	−0.02	0.04	0.02	0.04	−0.11	−0.06	0.05	−0.08	0.07	0.27 **	−0.68 **	−0.38 **	1	
18. PQOL	−0.12	0.20 **	−0.08	−0.00	−0.06	−0.06	0.05	0.07	−0.13	−0.05	0.12	−0.01	0.04	0.18 **	−0.49 **	−0.33 **	0.58 **	1

Note. *: *p* < 0.05; **: *p* < 0.01; BMI = body mass index; HbA_1_C = glycated hemoglobin; TG = triglyceride; TChol = total cholesterol; HDL-C = high density lipoprotein-cholesterol; LDL-C = low density lipoprotein-cholesterol; MMSE = Mini-Mental State Examination; ADL = activity of daily living; GDSs = geriatric depressive symptoms; SQ = sleep quality; PQOL, physical quality of life; MQOL, mental quality of life.

**Table 3:** Grey relational coefficient (GRC) (*n* = 220).

No	BMI	HbA_1_C	Fasting Sugar	TCholesterol	HDL-C	LDL-C	TG	SQ	MMSE	GDSs	PQOL	MQOL
1	0.8487	0.7561	0.4077	0.6774	0.8677	0.8581	0.3958	0.7500	0.3333	0.5714	0.7168	0.8410
2	0.8006	0.7887	0.4100	0.6752	1	0.8268	0.3967	0.8571	0.3333	0.5714	0.7297	0.8204
3	0.7709	0.7439	0.3967	0.6562	0.8235	0.7690	0.3811	0.8571	0.3333	0.5454	0.8571	0.8204
4	0.8059	0.7349	0.3904	0.7000	0.8467	0.8601	0.3964	0.8780	0.375	0.5714	0.7980	0.8375
5	0.8224	0.7593	0.4077	0.6961	0.8076	0.8383	0.4060	0.7500	0.3461	0.5454	0.9152	0.8589
6	0.8416	0.7530	0.4083	0.6695	0.8076	0.8212	0.3718	0.7500	0.3333	0.5217	0.6694	0.7671
7	0.8487	0.7469	0.3978	0.6428	0.8076	0.7469	0.3729	0.7346	0.5294	0.5000	0.7465	0.7790
8	0.8259	0.7500	0.4049	0.6535	0.7894	0.7690	0.3852	0.7826	0.3600	0.6666	0.9364	0.8305
9	0.8183	0.8169	0.4027	0.6759	0.7216	0.7823	0.5052	0.7659	0.6923	0.4800	0.7826	0.7730
10	0.7951	0.7787	0.3962	0.6774	0.7636	0.8325	0.3825	0.9473	0.4285	0.7500	0.8181	0.8973
11	0.8430	0.7689	0.3983	0.7175	0.8713	0.8194	0.3903	0.7659	0.3333	0.5217	0.7714	0.7790
12	0.8066	0.9384	0.7500	0.6468	0.7342	0.7658	0.4107	0.7826	0.3913	0.5714	0.7431	0.8204
13	0.8634	0.7721	0.4089	0.5855	0.7894	0.7658	0.3742	0.8780	0.4285	0.5454	0.8481	0.7913
14	0.8688	0.8280	0.4796	0.6596	0.7608	0.7891	0.4114	0.7200	0.375	0.4800	0.7980	0.7821
15	0.7744	0.7530	0.4100	0.6617	0.7806	0.8030	0.3838	0.7200	0.4285	0.5000	0.6612	0.7760
16	0.8169	0.8840	0.5240	0.6870	0.8641	0.8501	0.3652	0.7826	0.3333	0.5217	0.6835	0.7882
…	…	…	…	…	…	…	…	…	…	…	…	…
219	0.8270	0.7721	0.3962	0.7526	0.7553	0.7484	0.3789	0.7346	0.3461	0.5	0.6923	0.7701
220	0.8332	0.7593	0.3823	0.6468	0.7266	0.726	0.4344	0.9	0.3333	0.6666	0.8140	0.7730

Note. BMI = body mass index; HbA_1_C = glycated hemoglobin; TCholesterol = total cholesterol; HDL-C = high density lipoprotein-cholesterol; LDL-C = low density lipoprotein-cholesterol; TG = triglyceride; SQ = sleep quality; MMSE = Mini-Mental State Examination; GDSs = geriatric depressive symptoms; PQOL, physical quality of life; MQOL, mental quality of life.

## Data Availability

The data that support the findings of this study are available on request from the author Prof. C. I. Chen. The data are not publicly available due to privacy and ethical restrictions.

## Supplementary Materials

The following supporting information can be downloaded at: https://www.mdpi.com/article/10.3390/brainsci12121642/s1, Table S1. (Supplemental data). Grey relational coefficient (GRC) (*n* = 220).
