# Supplementary material for: Application of Grey Relational Analysis to Predict Dementia Tendency by Cognitive Function, Sleep Disturbances, and Health Conditions of Diabetic Patients
Source: Brain Sci. 2022 Nov 30;12(12):1642. doi: 10.3390/brainsci12121642 (PMC9775556; doi:10.3390/brainsci12121642)
Supplement: Supplementary file 1 [file brainsci-12-01642-s001.zip › brainsci-2036166-supplementary.pdf]

Table S1. (supplemental data). Grey relational coefficient (GRC) (*n* = 220)

No	BMI	HbA_1C	Sugar	TCholesteral	HDL_C	LDL_C	TG	SQ	MMSE	TGDS	PQOL	MQOL
1	0.8487	0.7562	0.4078	0.6774	0.8678	0.8582	0.3958	0.7500	0.3333	0.5714	0.7168	0.8410
2	0.8007	0.7888	0.4100	0.6752	1.0000	0.8269	0.3967	0.8571	0.3333	0.5714	0.7297	0.8204
3	0.7710	0.7439	0.3968	0.6563	0.8235	0.7691	0.3812	0.8571	0.3333	0.5455	0.8571	0.8204
4	0.8059	0.7349	0.3904	0.7000	0.8468	0.8602	0.3964	0.8780	0.3750	0.5714	0.7980	0.8375
5	0.8225	0.7593	0.4078	0.6961	0.8077	0.8383	0.4061	0.7500	0.3462	0.5455	0.9153	0.8590
6	0.8417	0.7531	0.4083	0.6695	0.8077	0.8213	0.3719	0.7500	0.3333	0.5217	0.6694	0.7672
7	0.8487	0.7469	0.3978	0.6429	0.8077	0.7469	0.3729	0.7347	0.5294	0.5000	0.7465	0.7791
8	0.8259	0.7500	0.4050	0.6535	0.7895	0.7691	0.3853	0.7826	0.3600	0.6667	0.9364	0.8306
9	0.8183	0.8170	0.4027	0.6760	0.7216	0.7823	0.5052	0.7660	0.6923	0.4800	0.7826	0.7731
10	0.7952	0.7787	0.3962	0.6774	0.7636	0.8326	0.3825	0.9474	0.4286	0.7500	0.8182	0.8973
11	0.8431	0.7689	0.3984	0.7175	0.8714	0.8194	0.3904	0.7660	0.3333	0.5217	0.7714	0.7791
12	0.8067	0.9385	0.7500	0.6468	0.7343	0.7658	0.4108	0.7826	0.3913	0.5714	0.7431	0.8204
13	0.8635	0.7722	0.4089	0.5855	0.7895	0.7658	0.3742	0.8780	0.4286	0.5455	0.8482	0.7913
14	0.8688	0.8281	0.4796	0.6597	0.7609	0.7891	0.4114	0.7200	0.3750	0.4800	0.7980	0.7821
15	0.7744	0.7531	0.4100	0.6618	0.7807	0.8031	0.3839	0.7200	0.4286	0.5000	0.6612	0.7761
16	0.8170	0.8841	0.5241	0.6870	0.8642	0.8501	0.3653	0.7826	0.3333	0.5217	0.6835	0.7882
17	0.8571	0.8592	0.4719	0.6796	0.7865	0.8521	0.3844	0.8780	0.3600	0.6000	0.8617	0.8105
18	0.8410	0.7531	0.3968	0.6488	0.7394	0.7908	0.3847	0.7660	0.3462	0.4800	0.7043	0.7913
19	0.8343	1.0000	0.4637	0.6468	0.7554	0.7674	0.3839	0.7200	0.3600	0.4800	0.7105	0.7882
20	0.8217	0.7787	0.3838	0.6402	0.7527	0.7578	0.3825	0.7059	0.4286	0.5000	0.7200	0.7913
21	0.7827	0.7821	0.3858	0.6590	0.8203	0.8176	0.3615	0.8571	0.3750	0.5455	0.7535	0.7913
22	0.8585	0.7531	0.4112	0.6840	0.7500	0.8832	0.3886	0.7500	0.3913	0.5000	0.7043	0.7821

No	BMI	HbA1C	Sugar	TCholesteral	HDL_C	LDL_C	TG	SQ	MMSE	TGDS	PQOL	MQOL
23	0.7662	0.7625	0.3952	0.6604	0.8203	0.7840	0.3688	0.8372	0.3462	0.5000	0.7200	0.7913
24	0.8497	0.7657	0.4509	0.6442	0.7609	0.7756	0.3833	0.7347	0.3913	0.8000	0.7902	0.9054
25	0.8927	0.8133	0.4242	0.6189	0.7554	0.8326	0.3985	0.7500	0.3462	0.5455	0.6807	0.7791
26	0.8420	0.7787	0.4182	0.6448	0.7394	0.7626	0.4143	0.7660	0.4737	0.6000	0.8351	0.8008
27	0.8126	0.7625	0.3915	0.6402	0.7554	0.7363	0.3747	0.8571	0.3462	0.5217	0.6894	0.7976
28	0.8519	0.7593	0.3946	0.7159	0.7778	0.8963	0.4086	0.7826	0.3333	0.6000	0.7500	0.9136
29	0.8576	0.8206	0.4078	0.6442	0.7266	0.7806	0.4006	0.7347	0.3750	0.5217	0.6835	0.7882
30	0.7972	0.7657	0.4016	0.6811	0.7664	0.8085	0.4136	0.7500	0.3462	0.5000	0.9474	0.8040
31	0.8077	0.8318	0.3925	0.6667	0.7749	0.7891	0.3747	0.7200	0.3462	0.5714	0.6864	0.7945
32	0.8132	0.8927	0.4434	0.6522	0.7985	0.7707	0.3867	0.8372	0.4091	0.5217	0.6835	0.7761
33	0.9294	0.7854	0.4200	1.0000	0.7343	0.8602	0.4055	0.8000	0.3333	0.5217	0.8617	0.7945
34	0.8257	0.7657	0.4044	0.6870	0.8607	0.8326	0.3742	0.7500	0.4286	0.7059	0.8020	0.9136
35	0.8514	0.7922	0.4095	0.6576	0.7865	0.8013	0.3693	0.7500	0.3462	0.4800	0.6694	0.7882
36	0.8717	0.7787	0.4336	0.6984	0.8108	0.6823	0.4421	0.8000	0.4500	0.5455	0.7364	0.7913
37	0.7832	0.7754	0.4112	0.6969	0.8607	0.8602	0.3630	0.7059	0.5625	0.5000	0.6750	0.7821
38	0.7883	0.7722	0.3978	0.6563	0.7598	0.8172	0.4055	0.7200	0.3333	0.5217	0.8710	0.8072
39	0.9176	0.7854	0.4095	0.6569	0.7778	0.7658	0.4079	0.7500	0.3333	0.5000	0.7043	0.7882
40	0.9399	0.7754	0.4170	0.6984	0.7778	0.8919	0.3988	0.7500	0.3600	0.5455	0.8571	0.7791
41	0.8680	0.8097	0.4317	0.7464	0.7749	0.7485	0.4146	0.6923	0.3333	0.5217	0.7043	0.7852
42	0.8643	0.7320	0.4011	0.6462	0.7368	0.7891	0.3777	0.7500	0.3333	0.4800	0.6807	0.7852
43	0.7960	0.7409	0.3925	0.6522	0.7778	0.7840	0.3761	0.7826	0.3333	1.0000	0.9101	0.9306
44	0.8468	0.7562	0.4106	0.6789	0.7636	0.8213	0.4172	0.7059	0.3600	0.5000	0.7043	0.7821

No	BMI	HbA$_1$C	Sugar	TCholesteral	HDL_C	LDL_C	TG	SQ	MMSE	TGDS	PQOL	MQOL
45	0.8103	0.7409	0.4061	0.7127	0.8171	0.9190	0.3779	0.7347	0.3462	0.5217	0.6807	0.7945
46	0.8093	0.7409	0.3915	0.6225	0.7749	0.7132	0.3680	0.8000	0.6000	0.5000	0.6585	0.7701
47	0.8408	0.7439	0.3952	0.6855	0.8367	0.8213	0.3673	0.7347	0.3462	0.5000	0.6694	0.7761
48	0.8799	0.7439	0.4078	0.6576	0.7636	0.8049	0.3732	0.7200	0.3750	0.4800	0.7168	0.7852
49	0.7964	0.7409	0.4123	0.6818	0.7473	0.8176	0.4443	0.7826	0.3333	0.5455	0.7431	0.7913
50	0.7938	0.8927	0.6563	0.7412	0.7292	0.8789	0.5785	0.7826	0.3333	0.5000	0.7137	0.7882
51	0.8445	0.7821	0.4343	0.6481	0.7527	0.7823	0.3665	0.8182	0.3462	0.5714	0.7074	0.8238
52	0.8646	0.7625	0.4158	0.6604	0.8077	0.7823	0.3795	0.7660	0.3333	0.5217	0.6835	0.7945
53	0.8355	0.7500	0.3952	0.6863	0.7836	0.8663	0.3742	0.6923	0.6923	0.5455	0.7465	0.7791
54	0.8134	0.8356	0.4249	0.6502	0.7609	0.7773	0.3833	0.7500	0.3333	0.4800	0.6864	0.7556
55	0.8422	0.7689	0.4200	0.6938	0.8300	0.8541	0.3758	0.8182	0.3462	0.8571	0.8100	0.8701
56	0.8210	0.7500	0.4055	0.6863	0.8203	0.8462	0.3825	0.8780	0.3333	0.7500	0.7941	0.8627
57	0.9409	0.7439	0.4055	0.6549	0.8502	0.7578	0.3670	0.8571	0.3333	0.5455	0.7297	0.7945
58	0.8716	0.7531	0.4118	0.6442	0.7554	0.7547	0.4083	0.8571	0.3333	0.5217	0.7265	0.8105
59	0.8308	0.7722	0.3952	0.6597	0.7554	0.7978	0.3909	0.8182	0.3333	0.8000	0.8223	0.8894
60	0.8330	0.7821	0.4261	0.6961	0.8046	0.8643	0.4146	0.7660	0.3333	0.5217	0.6694	0.7976
61	0.8191	0.8592	0.4949	0.6377	0.7447	0.7363	0.4279	0.8000	0.3333	0.5000	0.6894	0.7882
62	0.7992	0.7379	0.3899	0.6351	0.7500	0.7531	0.3721	0.7347	0.3913	0.5455	0.6807	0.7761
63	0.8141	0.7888	0.4089	0.7159	0.7554	0.8231	0.4755	0.7347	0.3750	0.5000	0.6694	0.7791
64	0.8273	0.7439	0.3889	0.6528	0.8861	0.7319	0.3630	0.8571	0.3600	0.5217	0.7168	0.7731
65	0.7944	0.7409	0.4011	0.6716	0.7500	0.7891	0.4272	0.6923	0.5294	0.5000	0.7013	0.7731
66	0.8443	0.7500	0.4100	0.7683	0.7447	0.8250	0.9745	0.7347	0.3333	0.5000	0.6983	0.7672

No	BMI	HbA_1C	Sugar	TCholesteral	HDL_C	LDL_C	TG	SQ	MMSE	TGDS	PQOL	MQOL
67	0.8435	0.7531	0.4100	0.6716	0.7955	0.8288	0.3825	0.7059	0.3462	0.5217	0.6864	0.7761
68	0.8043	0.7625	0.4158	0.6583	0.7636	0.7961	0.3895	0.7826	0.3462	0.5455	0.7330	0.7731
69	0.7710	0.7593	0.4123	0.6908	0.8015	0.8602	0.3850	0.8372	0.3333	0.5455	0.7980	0.8105
70	0.8107	0.7922	0.4033	0.6502	0.7664	0.7723	0.3875	0.7826	0.4091	0.5000	0.6667	0.7761
71	0.8190	0.7469	0.4044	0.6855	0.7985	0.8602	0.3698	0.7347	0.3462	0.4800	0.6694	0.7731
72	0.8298	0.7531	0.4044	0.6833	0.7581	0.8622	0.4006	0.7660	0.4091	0.5455	0.7137	0.7791
73	0.8009	0.7821	0.4292	0.6357	0.7447	0.7516	0.4000	0.7826	0.3333	0.5000	0.6532	0.7701
74	0.7754	0.7409	0.3904	0.6818	0.8300	0.8213	0.3737	0.7347	0.3600	0.5000	0.6722	0.7701
75	0.7892	0.7562	0.3915	0.6840	0.7985	0.8176	0.3861	0.6923	0.7500	0.5455	0.7297	0.7852
76	0.8181	0.8206	0.3858	0.6840	0.7925	0.8250	0.4623	0.7059	0.4091	0.5217	0.6807	0.7791
77	0.8699	0.7821	0.4330	0.6300	0.7527	0.7469	0.3895	0.7347	0.3600	0.5000	0.6953	0.7761
78	0.8569	0.7722	0.4273	0.6468	0.7368	0.7823	0.3915	0.9474	0.3750	0.5217	0.7751	0.7976
79	0.8881	0.7469	0.3915	0.6535	0.8015	0.7531	0.3872	0.7500	0.3913	0.5000	0.7714	0.7913
80	0.8780	0.7469	0.3995	0.6900	0.7807	0.8139	0.4152	0.7500	0.3600	0.5217	0.7105	0.7852
81	0.8117	0.7593	0.3995	0.6306	0.7420	0.7423	0.3803	0.6923	0.3600	0.5000	0.6694	0.7731
82	0.8399	0.7689	0.4224	0.6632	0.7420	0.7943	0.4061	0.7200	0.4500	0.5000	0.6667	0.7731
83	0.8035	0.8281	0.3904	0.6646	0.7955	0.7978	0.3787	0.7660	0.5294	0.5714	0.6983	0.7913
84	0.7971	0.8433	0.4925	0.6767	0.8077	0.8013	0.3970	0.7200	0.3333	0.5455	0.6835	0.7701
85	1.0000	0.9015	0.4788	0.6818	0.7581	0.8231	0.3785	0.8372	0.3600	0.5000	0.7232	0.7701
86	0.8913	0.7291	0.3930	0.6674	0.8678	0.7691	0.3779	0.7500	0.4091	0.4800	0.7714	0.7761
87	0.8127	0.7657	0.4135	0.6488	0.7664	0.7874	0.3660	0.7826	0.4286	0.5217	0.8223	0.7976
88	0.8183	0.7531	0.4362	0.7554	0.7368	1.0000	0.4648	0.7500	0.4286	0.5455	0.6894	0.7913

No	BMI	HbA_1C	Sugar	TCholesteral	HDL_C	LDL_C	TG	SQ	MMSE	TGDS	PQOL	MQOL
89	0.8576	0.7500	0.4011	0.6646	0.7865	0.7840	0.3944	0.7347	0.3333	0.5000	0.6864	0.7945
90	0.8330	0.8026	0.3989	0.6231	0.7317	0.7363	0.3777	0.7347	0.3333	0.5000	0.6983	0.7791
91	0.8059	0.7439	0.4078	0.6468	0.8077	0.7610	0.3678	0.7347	0.4091	0.5455	0.6835	0.7761
92	0.8754	0.7500	0.3925	0.6618	0.7500	0.7996	0.4012	0.7660	0.3333	0.5217	0.6667	0.7821
93	0.7817	0.8133	0.4170	0.6681	0.7985	0.7806	0.3643	0.7059	0.4737	0.5217	0.6835	0.7731
94	0.7988	0.7722	0.3978	0.6535	0.8171	0.7594	0.3878	0.8182	0.5294	0.5455	0.6894	0.7791
95	0.8787	0.8632	0.4421	0.6549	0.7865	0.7363	0.4395	0.7826	0.3600	0.5000	0.7043	0.7643
96	0.8605	0.7593	0.4141	0.6667	0.7636	0.8383	0.3698	0.7347	0.4286	0.5000	0.6750	0.7791
97	0.9075	0.8026	0.4249	0.6604	0.7581	0.8031	0.3870	0.8571	0.4286	0.6000	0.7397	0.7913
98	0.8919	0.8318	0.4279	0.6468	0.7836	0.7642	0.3753	0.8000	0.3333	0.6316	0.7642	0.7821
99	0.8335	0.7379	0.3910	0.6667	0.7778	0.8176	0.3693	0.8182	0.3913	0.5455	0.6694	0.7976
100	0.8165	0.7262	0.3843	0.6731	0.8642	0.7908	0.3719	0.8182	0.3333	0.5217	0.6694	0.8627
101	0.8411	0.7754	0.4000	0.6455	0.7554	0.7348	0.4356	0.8000	1.0000	0.8571	0.8141	0.9901
102	0.8175	0.7176	0.3910	0.6583	0.7836	0.8049	0.3630	0.8372	0.4286	0.5217	0.6983	0.7528
103	0.8092	0.7821	0.3707	0.6984	0.8434	0.8501	0.3793	0.7660	0.3600	0.5217	0.6983	0.7945
104	0.8017	0.7821	0.3957	0.6724	0.8108	0.8176	0.3716	0.8571	0.3333	0.5217	0.8663	0.9178
105	0.8499	0.7562	0.4011	0.6535	0.7865	0.7790	0.3761	0.8000	0.3600	0.5217	0.7137	0.8701
106	0.7972	0.7349	0.4158	0.6402	0.8015	0.7319	0.3742	0.8780	0.3462	0.5217	0.7074	0.7731
107	0.7910	0.7625	0.4170	0.6488	0.7925	0.7691	0.3688	0.9474	0.3600	0.6000	0.6953	0.8340
108	0.8028	0.7854	0.4027	0.6597	0.7749	0.7790	0.3847	0.7826	0.3600	0.5455	0.6894	0.7882
109	0.8016	0.7593	0.3889	0.6724	0.7447	0.8288	0.3929	0.8000	0.4737	0.5714	0.7570	0.8445
110	0.7781	0.7379	0.3813	0.6709	0.7266	0.8326	0.3803	0.7826	0.3600	0.5714	0.8394	0.9136

No	BMI	HbA_1C	Sugar	TCholesteral	HDL_C	LDL_C	TG	SQ	MMSE	TGDS	PQOL	MQOL
111	0.8526	0.7625	0.4382	0.6435	0.7554	0.7840	0.4668	0.8000	0.3333	0.6000	0.8757	0.9526
112	0.8672	0.7379	0.3863	0.6688	0.7500	0.8213	0.4245	0.7826	0.3600	0.5455	0.8020	0.8816
113	0.8321	0.7689	0.4230	0.6287	0.7527	0.7289	0.3973	0.7200	0.3600	0.5000	0.7751	0.7761
114	0.8190	0.7854	0.3978	0.6364	0.7581	0.7363	0.4045	0.9231	0.3600	0.5714	0.6894	0.8040
115	0.9086	0.7593	0.4100	0.6549	0.7447	0.7926	0.3878	0.8000	0.3600	0.6000	1.0000	0.9349
116	0.8304	0.8097	0.4421	0.6833	0.7955	0.8541	0.3753	0.7059	0.3333	0.5217	0.6750	0.7821
117	0.8687	0.7349	0.4044	0.6604	0.7955	0.7874	0.3878	0.7500	0.3462	0.5217	0.6667	0.7913
118	0.7772	0.7320	0.3838	0.6760	0.8268	0.8345	0.3761	0.7059	0.5000	0.5455	0.6953	0.7731
119	0.8096	0.8472	0.4667	0.6660	0.8537	0.7707	0.3872	0.6923	0.3600	0.5455	0.6585	0.7672
120	0.8148	0.7754	0.4066	0.6515	0.8046	0.7516	0.3630	0.8000	0.3600	0.5455	0.6953	0.7913
121	0.8086	0.8026	0.4038	0.6219	0.7749	0.7188	0.3615	0.7200	0.3462	0.5217	0.6778	0.7761
122	0.8569	0.7291	0.3936	0.6660	0.7664	0.7978	0.3898	0.7500	0.3750	0.4800	0.6778	0.7731
123	0.8147	0.7500	0.3989	0.6409	0.7343	0.7348	0.3690	0.7200	0.3913	0.5217	0.7043	0.7643
124	0.8101	0.7689	0.4147	0.6885	0.7554	0.7978	0.3997	0.8780	0.4737	0.8000	0.9205	0.9663
125	0.8706	0.7722	0.4388	0.6409	0.7664	0.7610	0.3926	0.8182	0.3333	0.5000	0.8351	0.7913
126	0.8378	0.7148	0.4066	0.6674	0.7721	0.8176	0.3719	0.7500	0.3333	0.5000	0.6694	0.7731
127	0.9456	0.7409	0.3946	0.6448	0.7581	0.7740	0.3842	0.8571	0.4737	0.5217	0.6750	0.7945
128	0.8173	0.7500	0.4005	0.6803	0.8367	0.8326	0.3675	0.7347	0.3462	0.5000	0.6585	0.7643
129	0.7975	0.7469	0.3978	0.6542	0.8300	0.7642	0.3633	0.8182	0.3913	0.5000	0.6953	0.7791
130	0.8185	0.7888	0.3930	0.6931	0.8046	0.7806	0.3964	0.8182	0.3462	0.5000	0.7941	0.7945
131	0.9233	0.8433	0.4153	0.6275	0.7807	0.7304	0.3719	0.7660	0.3600	0.5714	0.7902	0.8816
132	0.8320	0.7531	0.3904	0.6422	0.7721	0.7658	0.3693	0.8182	0.6000	0.5217	0.7826	0.8171

No	BMI	HbA_1C	Sugar	TCholesteral	HDL_C	LDL_C	TG	SQ	MMSE	TGDS	PQOL	MQOL
133	0.8000	0.7722	0.4095	0.6495	0.7317	0.7926	0.3806	0.7500	0.3750	0.5000	0.7397	0.7701
134	0.8412	0.8318	0.4212	0.6522	0.7836	0.7691	0.3961	0.7500	0.3333	0.5000	0.6667	0.7672
135	0.8095	0.7320	0.3904	0.6294	0.7420	0.7454	0.3782	0.8000	0.3462	0.4800	0.7105	0.7672
136	0.7630	0.8472	0.3750	0.6625	0.7368	0.7996	0.3964	0.9000	0.5294	0.5714	0.7105	0.8105
137	0.7980	0.7176	0.3973	0.6000	0.7609	0.7874	0.3594	0.7200	0.4737	0.5000	0.6559	0.7672
138	0.7635	0.7722	0.3989	0.6495	0.8203	0.7626	0.3653	0.7826	0.3333	0.5217	0.6983	0.7672
139	0.8258	0.7922	0.3858	0.6522	0.8642	0.7531	0.3638	0.7826	0.3333	0.5217	0.6750	0.7945
140	0.8133	0.7991	0.3952	0.6796	0.8268	0.7840	0.3708	0.7826	0.3462	0.5217	0.7074	0.7791
141	0.8842	0.7593	0.4022	0.6716	0.8333	0.7740	0.3703	0.8182	0.3600	0.5455	0.6953	0.8072
142	0.8374	0.7625	0.4349	0.6481	0.7836	0.7674	0.3787	0.7500	0.4737	0.6316	0.7168	0.8340
143	0.8045	0.7531	0.4044	0.6674	0.7664	0.7857	0.4293	0.8372	0.3462	0.5714	0.6953	0.7791
144	0.8502	0.7531	0.3863	0.6364	0.7778	0.7500	0.3721	0.7826	0.3333	0.4800	0.6722	0.7761
145	0.9059	0.8243	0.4368	0.6893	0.8140	0.8705	0.3734	0.8182	0.3913	0.5000	0.6835	0.8040
146	0.8796	0.7854	0.4182	0.6660	0.7500	0.7891	0.4036	0.7500	0.3462	0.5217	0.7043	0.7643
147	0.8980	0.7409	0.3925	0.6639	0.8077	0.7926	0.3711	0.8372	0.4500	0.5455	0.8617	0.7791
148	0.8297	0.7439	0.3899	0.6542	0.7721	0.8013	0.3777	0.7347	0.3750	0.4800	0.6807	0.7791
149	0.7753	0.7439	0.3936	0.6569	0.7554	0.7961	0.3875	0.7347	0.3333	0.5217	0.7788	0.7528
150	0.8502	0.7439	0.4129	0.6409	0.7343	0.7806	0.3785	0.7826	0.3462	0.5217	0.6585	0.7791
151	0.8570	0.7562	0.4016	0.6900	0.8468	0.8541	0.3685	0.7660	0.3462	0.5000	0.6807	0.7701
152	0.7819	0.7593	0.4078	0.6724	0.7581	0.8422	0.3795	0.7660	0.3462	0.5000	0.6923	0.7585
153	0.7927	0.7562	0.4038	0.6569	0.7925	0.7806	0.3867	0.7660	0.3333	0.5000	0.6835	0.7643
154	0.8328	0.7562	0.4236	0.6549	0.7807	0.7485	0.3909	0.7200	0.4500	0.5217	0.7232	0.7672

No	BMI	HbA_1C	Sugar	TCholesteral	HDL_C	LDL_C	TG	SQ	MMSE	TGDS	PQOL	MQOL
155	0.8610	0.9150	0.4579	0.6796	0.7865	0.7773	0.3844	0.8571	0.3462	0.5217	0.7788	0.8171
156	0.8243	0.8281	0.4055	0.6442	0.7749	0.7723	0.3693	0.8372	0.3462	0.5000	0.6612	0.7761
157	0.8777	0.8318	0.3995	0.6495	0.7749	0.7691	0.3739	0.8571	0.3462	0.5000	0.7535	0.8272
158	0.8737	0.8243	0.4401	0.6840	0.8140	0.7891	0.3703	0.8000	0.3333	0.5000	0.7043	0.7821
159	0.8091	0.7754	0.4401	0.6256	0.7343	0.7348	0.3861	0.7500	0.3333	0.5000	0.7941	0.8664
160	0.8120	0.8927	0.5052	0.7464	0.7985	0.7531	0.4341	0.7500	0.3462	0.5455	0.6835	0.7882
161	0.8153	0.7689	0.4038	0.7031	0.8015	0.8013	0.3921	0.8182	0.4500	0.5217	0.6778	0.7821
162	0.8008	0.8133	0.4468	0.6745	0.7636	0.8013	0.3856	0.7826	0.4286	0.5217	0.8141	0.7643
163	0.8393	0.7469	0.4033	0.6674	0.8400	0.7891	0.3785	0.7500	0.3600	0.5217	0.7714	0.7852
164	0.8789	0.7562	0.4055	0.6716	0.8434	0.7610	0.3645	0.7660	0.3333	0.5217	0.6750	0.7761
165	0.8212	0.7320	0.3838	0.6893	0.8015	0.8442	0.3822	0.8182	0.3333	0.5000	0.6807	0.7701
166	0.8169	0.7593	0.4100	0.6306	0.7636	0.7547	0.3623	0.7200	0.3913	0.5217	0.6807	0.7791
167	0.8169	0.7991	0.4408	0.6597	0.7609	0.8067	0.3761	0.7500	0.3913	0.5000	0.6807	0.7701
168	0.8860	0.7922	0.4095	0.6170	0.7216	0.7393	0.3648	0.8571	0.3750	0.7059	0.8438	0.8664
169	0.8353	0.7625	0.3952	0.6306	0.8015	0.7333	0.3668	0.7660	0.5625	0.5455	0.7297	0.7761
170	0.8469	0.8026	0.4129	0.7258	0.7664	0.9553	0.4248	0.6923	0.3600	0.5455	0.6778	0.7701
171	0.8585	0.7787	0.4123	0.7127	0.7865	0.9332	0.3915	0.7200	0.3462	0.5217	0.6667	0.8105
172	0.7975	0.7722	0.4200	0.6495	0.8203	0.7723	0.3678	0.7826	0.4091	0.5455	0.6778	0.7791
173	0.8798	0.8206	0.4182	0.6442	0.7500	0.7563	0.4130	0.7200	0.3462	0.5000	0.6585	0.7672
174	0.8052	0.7233	0.4286	0.6415	0.7664	0.7594	0.3904	0.7059	0.3462	0.5000	0.8394	0.7731
175	0.8602	0.7291	0.3930	0.6415	0.7807	0.7642	0.3793	0.7347	0.4091	0.4800	0.7074	0.7731
176	0.7867	0.8097	0.4005	0.6448	0.8434	0.7304	0.3719	0.7200	0.4500	0.5217	0.6953	0.7761

No	BMI	HbA_1C	Sugar	TCholesteral	HDL_C	LDL_C	TG	SQ	MMSE	TGDS	PQOL	MQOL
177	0.8262	0.8356	0.4368	0.6542	0.7721	0.7961	0.3750	0.7200	0.3333	0.5217	0.6953	0.7731
178	0.8462	0.7379	0.4066	0.6639	0.7368	0.8250	0.3970	0.7059	0.3750	0.5000	0.6585	0.7500
179	0.8093	0.7821	0.3962	0.7360	0.7292	0.7217	0.7177	0.7500	0.3462	0.7059	0.7980	0.7852
180	0.8666	0.8206	0.3910	0.6396	0.7985	0.7454	0.3724	0.7826	0.3600	0.5455	0.6835	0.8204
181	0.8445	0.7722	0.4089	0.6415	0.7807	0.7531	0.3668	0.7500	0.3913	0.5217	0.6667	0.7672
182	0.8467	0.7349	0.4000	0.6442	0.7581	0.7516	0.4089	0.8780	0.4500	0.8000	0.8617	0.9136
183	0.8411	0.8756	0.6151	0.6364	0.7527	0.7304	0.4079	0.8372	0.3750	0.5455	0.7500	0.7731
184	0.8715	0.7562	0.4055	0.6515	0.8235	0.7578	0.3726	0.7500	0.3333	0.5217	0.6807	0.7821
185	0.8486	0.8394	0.4579	0.8836	0.7581	0.8067	1.0000	0.7826	0.3750	0.7059	0.6983	0.8445
186	0.7901	0.7262	0.3828	0.6468	0.8571	0.7485	0.3650	0.7200	0.4737	0.5000	0.6835	0.7672
187	0.8417	0.7821	0.3853	0.6370	0.7581	0.7423	0.3833	0.7660	0.3333	0.5000	0.6864	0.7731
188	0.8476	0.8243	0.4298	0.6639	0.7778	0.7978	0.4205	0.8571	0.3462	0.5000	0.7535	0.8933
189	0.9271	0.8133	0.4147	0.6716	0.7836	0.8288	0.3732	0.7200	0.3333	0.5217	0.6953	0.7672
190	0.8060	0.7957	0.4273	0.6475	0.7292	0.7806	0.4006	0.9231	0.3600	0.5714	0.6953	0.7852
191	0.8653	0.7262	0.3941	0.6495	0.8140	0.7642	0.3688	0.8571	0.3333	0.5455	0.7570	0.7672
192	0.8476	0.8394	0.4055	0.6893	0.7836	0.8231	0.4599	0.7347	0.3333	0.5217	0.6983	0.7672
193	0.8441	0.7320	0.3874	0.6646	0.8607	0.7790	0.3650	0.8000	0.3462	0.6316	0.7465	0.8040
194	0.8084	0.7469	0.3984	0.6811	0.7985	0.8422	0.3892	0.8372	0.3333	0.5000	0.6983	0.7614
195	0.9416	0.7531	0.4147	0.6313	0.7692	0.7469	0.3747	0.8372	0.3462	0.5000	0.7265	0.7701
196	0.8411	0.8133	0.5475	0.6535	0.7865	0.7578	0.3795	0.7500	0.3462	0.5217	0.6559	0.7701
197	0.8300	0.7349	0.3973	0.6597	0.8434	0.7756	0.3711	0.8780	0.3462	0.6667	0.6585	0.7945
198	0.8106	0.8133	0.4106	0.6639	0.7985	0.8031	0.3606	0.7347	0.3600	0.5000	0.6694	0.7701

No	BMI	HbA1C	Sugar	TCholesteral	HDL_C	LDL_C	TG	SQ	MMSE	TGDS	PQOL	MQOL
199	0.8228	0.8062	0.3936	0.6542	0.7368	0.7756	0.4241	0.9231	0.3913	0.7059	0.8852	1.0000
200	0.8295	0.7531	0.3984	0.6653	0.7527	0.8194	0.3769	0.7500	0.3462	0.5217	0.6983	0.7731
201	0.8483	0.8243	0.4147	0.6287	0.7664	0.7289	0.3716	0.7200	0.3333	0.5455	0.6894	0.7731
202	0.8238	0.7469	0.4129	0.6569	0.8046	0.7723	0.3755	0.7826	0.3462	0.5217	0.8482	0.7672
203	0.8060	0.7593	0.3973	0.6325	0.8140	0.7578	0.3769	0.7660	0.3600	0.5217	0.6894	0.7701
204	0.8345	0.7500	0.3984	0.5888	0.7985	0.9404	0.3870	0.8182	0.3333	0.4800	0.6864	0.7821
205	0.7830	0.7439	0.3962	0.6954	0.7925	0.8919	0.3753	0.7660	0.3600	0.5217	0.7826	0.8481
206	0.8835	0.7957	0.4551	0.6625	0.8400	0.7857	0.3795	0.7500	0.3333	0.5217	0.8710	0.7701
207	0.8005	0.7957	0.4176	0.6646	0.8235	0.8013	0.3678	0.7500	0.4091	0.5000	0.6953	0.7821
208	0.8224	0.7262	0.3848	0.7394	0.8537	0.9213	0.4680	1.0000	0.4500	0.8000	0.7570	0.8445
209	0.7720	0.7722	1.0000	0.6632	0.7527	0.7610	0.4824	0.9000	0.3333	0.6667	0.8571	0.8072
210	0.7946	0.8356	0.4544	0.6660	0.7925	0.7485	0.3915	0.7660	0.4091	0.5217	0.6953	0.7761
211	0.8168	0.7409	0.4530	0.6528	0.7420	0.8013	0.4092	0.8000	0.3333	0.6000	0.7606	0.7882
212	0.7997	0.7562	0.4011	0.6760	0.8678	0.8213	0.3650	0.8780	0.3462	0.5000	0.6983	0.7821
213	0.8375	0.7469	0.4044	0.6569	0.7609	0.8085	0.3713	0.7826	0.3462	0.5000	0.7200	0.7731
214	0.7721	0.7562	0.3973	0.6893	0.8140	0.7439	0.3763	0.8000	0.3462	0.5000	0.6778	0.7791
215	0.8181	0.7409	0.4055	0.6781	0.7473	0.8941	0.3761	0.7826	0.3600	0.5217	0.7902	0.7791
216	0.7950	0.8026	0.4530	0.6760	0.7636	0.7469	0.3944	0.7500	0.3462	0.4800	0.7364	0.7945
217	0.8097	0.8318	0.4089	0.6583	0.8015	0.7943	0.3793	0.8000	0.3462	0.5455	0.8182	0.8138
218	0.9377	0.9337	0.5131	0.6415	0.7420	0.7943	0.3777	0.7500	0.3333	0.5217	0.7043	0.7821
219	0.8271	0.7722	0.3962	0.7527	0.7554	0.7485	0.3790	0.7347	0.3462	0.5000	0.6923	0.7701
220	0.8332	0.7593	0.3823	0.6468	0.7266	0.7260	0.4345	0.9000	0.3333	0.6667	0.8141	0.7731

Note. BMI= Body Mass Index; HbA_1C = Glycated hemoglobin; TCholesterol=Total Cholesterol; HDL_C = High Density Lipoprotein-Cholesterol ; LDL_C= Low Density Lipoprotein-Cholesterol; TG= Triglyceride; SQ = Sleep Quality; MMSE= Mini-Mental Status Examination; GDSs= Geriatric Depressive symptoms; PQOL; Physical quality of life; MQOL, Mental quality of lif
